# Metastatic Sarcomatoid Squamous Cell Carcinoma of the Cervix Presenting with Chest Mass

**DOI:** 10.1155/2017/5264564

**Published:** 2017-09-14

**Authors:** Lilit Karapetyan, Manoj Rai, Om Dawani, Heather S. Laird-Fick

**Affiliations:** ^1^Michigan State University Department of Medicine and EW Sparrow Hospital, 804 Service Rd, Room B301, East Lansing, MI 48824, USA; ^2^Michigan State University Department of Medicine, 804 Service Rd, Room B316, East Lansing, MI 48824, USA

## Abstract

**Background:**

Sarcomatoid squamous cell carcinoma is a rare and aggressive form of cervical cancer. We report a case of metastatic sarcomatoid squamous cell carcinoma (SSCC) of cervix that presented with an anterior chest wall mass.

**Case:**

A 43-year-old Hispanic female presented with a two-month history of a central chest wall mass. The patient's only past medical history was SSCC of the cervix, stage IIB, diagnosed two years priorly. She underwent neoadjuvant chemoradiation therapy (CRT) with cisplatin followed by radical hysterectomy. Surgical margins were positive which led to adjuvant CRT with carboplatin and paclitaxel. PET scan 4 months after the postoperative treatment was negative for recurrence and metastatic disease. On current presentation, the CT chest revealed anterior mediastinal destructive soft tissue mass involving sternum, and the biopsy showed SSCC. The patient received palliative radiation therapy to her chest with improvement in pain and ability to swallow. After discussing the prognosis she refused further chemotherapy and decided on hospice care.

**Conclusion:**

Despite good response to first-line therapy, SSCC tends to recur early and does not respond to second-line therapy. Radiation therapy seems to be the most effective modality for treatment, but randomized controlled trials of therapy are impractical.

## 1. Introduction

Cervical cancer is the fourth most common cancer in women worldwide, causing approximately 270,000 deaths per year. Widespread use of cervical cancer screening has reduced morbidity and mortality in the United States, but in 2017 an estimated 12,900 cases and 4,120 deaths are still expected. Racial and ethnic disparities also persist, with incidence remaining high among Hispanic women [1–3].

The squamous cell histologic subtype accounts for 80% of cervical cancer cases and is classically preceded by precancerous changes detectable by Pap smear. Adenocarcinoma of the cervix behaves similarly, although it develops from the endocervix rather than the transitional zone. Both subtypes of cervical cancer tend to be locally aggressive, with metastases primarily to lymph nodes, lung, and liver [2].

In contrast, sarcomatoid squamous cell carcinoma of the cervix (SSCC) is an extremely rare and aggressive variant, with similar sites of metastasis. There are no well-established guidelines for its treatment; most cases are managed similar to squamous cell carcinoma [3–6].

We report a case of metastatic SSCC of the cervix that presented with an anterior chest wall mass, an unusual site of spread for this rare tumor.

## 2. Case Presentation

**Figure 1:**
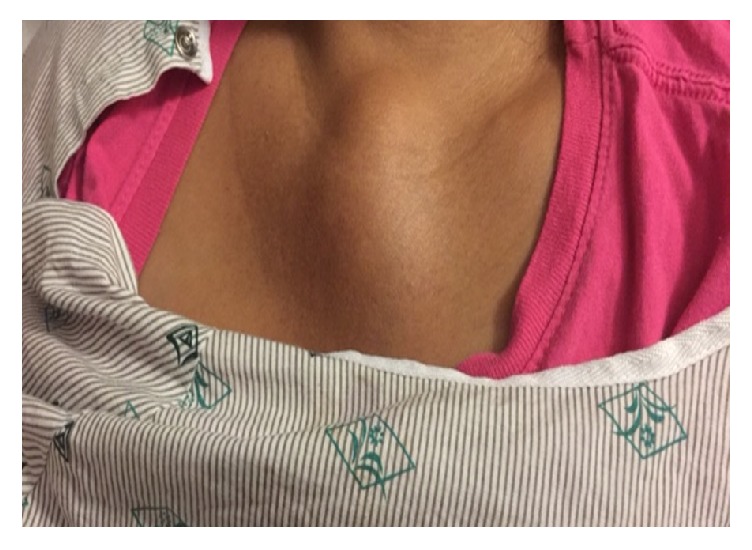
Anterior central chest wall mass.

A 43-year-old G6P6 Hispanic female presented with a two-month history of a central chest wall mass (Figure 1). The patient's only past medical history was SSCC of the cervix, stage IIB, diagnosed two years priorly. At that time, she presented with severe vaginal bleeding. Transvaginal and transabdominal ultrasounds showed abnormal enlargement of the uterine cervix. A CT abdomen/pelvis confirmed a heterogenous cervical mass without evidence of metastatic disease. Histopathology revealed SSCC of the cervix, positive for human papilloma virus (HPV). The patient was clinically staged as International Federation of Gynecology and Obstetrics (FIGO) stage IIb and subsequently started on chemoradiation therapy with weekly cisplatin and external beam radiation therapy totaling 4140 cGy before undergoing radical hysterectomy. She had microscopically positive margins which led to postoperative adjuvant chemoradiation therapy with carboplatin and paclitaxel. PET scan four months after treatment was negative for recurrent or metastatic disease. She was subsequently lost to follow-up because of lack of insurance and a language barrier.

**Figure 2:**
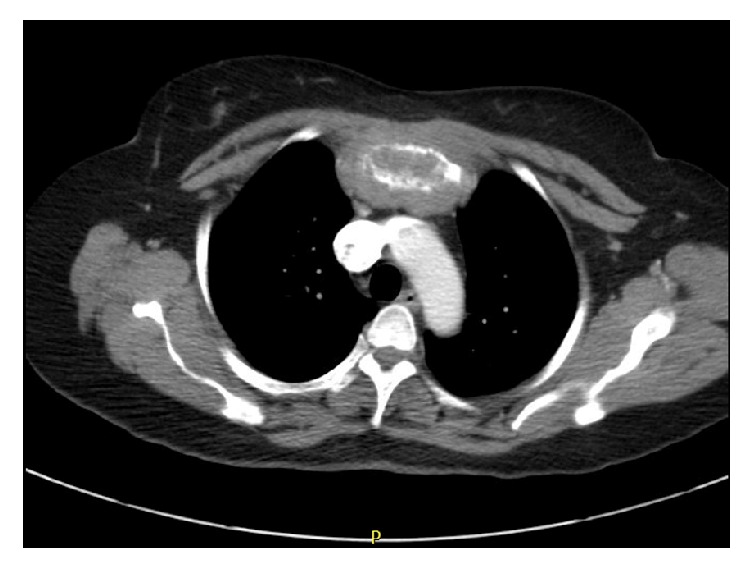
2.1 × 1.5 cm soft tissue infiltrating along the manubrium with underlying destruction of the bone.

**Figure 3:**
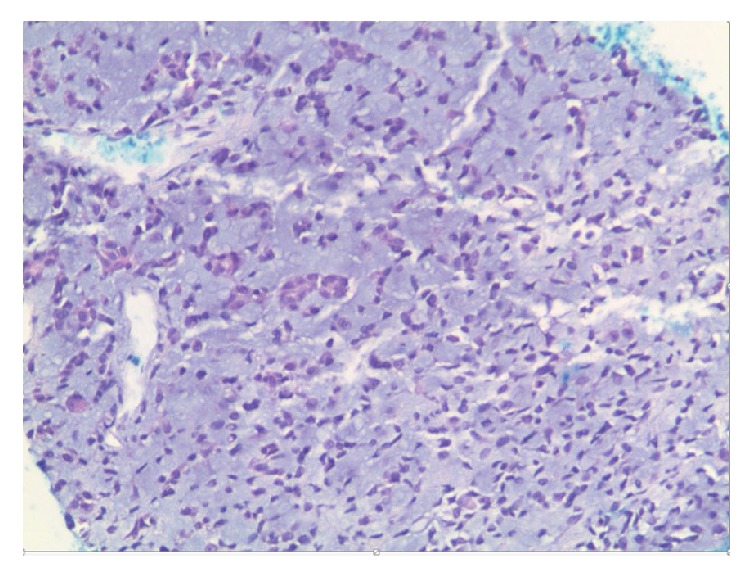
HE staining of tumor showing highly cellular epithelial cells with fascicles of short spindle cells.

She again sought medical attention only when she experienced intolerable pain and difficulty in swallowing associated with enlargement of a new mass on her chest (Figure 1). CT chest/abdomen/pelvis and MRI brain/cervical spine showed extensive infiltration by a soft tissue mass, associated bony destruction, and pulmonary nodules (Figure 2). The soft tissue mass extended into the anterior mediastinum, the right upper cervical canal, and neural foramena at the levels of C2–C4 and into the paraspinal muscles at the level of C3. No cord compression was noted. CT-guided core biopsy revealed tumor predominantly composed of spindle cells (Figure 3) with features of SSCC, immunoreactivity for cytokeratin (AE1/AE3), CAM5.2, and vimentin, and weakly positive for muscle actin, S100, and p63. The patient received five palliative radiation treatments to her chest with improvement in pain and swallowing. After discussion of the benefits and risks of palliative chemotherapy, she opted for hospice care.

## 3. Discussion

Sarcomatoid carcinoma tends to affect the upper aerodigestive tract (i.e., larynx, pharynx, and esophagus) and skin. It comprises only 1-2% of all gynecological malignancies [7]. A comprehensive literature review using the terms “sarcomatoid carcinoma” and “squamous cell carcinoma” identified 20 reported cases, including a case series of nine patients (Brown et al.) [4–6, 8–10]. As with the more common types of cervical cancer, HPV is the primary etiologic factor identified to date; high risk subtypes 16 and 18 have been found in both squamous and sarcomatoid components of the tumor [11].

Two hypotheses exist regarding the development of the sarcomatoid features. In an esophageal model, researchers have described transformation from squamous cell to spindle cell cancer with loss of epithelial cells from the basal layer of the tissue. The spindle cells contain desmosomes and tonofilaments, supporting their squamous cell origin. Alternatively, the carcinoma could be a chimera of squamous and spindle cell components arising from two different stem cell lines [7, 12].

As for the more common subtypes, sarcomatoid tumors are more common with increasing age. The median age at presentation is 67 years. Women are generally symptomatic at the time of diagnosis, with abnormal vaginal bleeding or foul smelling discharge. Cervical lesions are readily visible on physical examination.

Histology demonstrates the pathognomonic squamous and spindle cell components. Immunohistochemistry is usually positive for mesenchymal and epithelial components like cytokeratin and vimentin [7, 12, 13].

The FIGO system is used for staging of the disease. In the absence of evidence for this histologic subtype, most women receive treatments based on the recommendations for squamous cervical carcinoma. Surgery is the preferred choice of treatment for early stage disease. Tumor size, margin status, and tumor biology guide the need for adjuvant radiation therapy after resection for local prevention of recurrence.

Prognosis of SSCC of the cervix, however, is more similar to sarcoma than other types of cervical cancer. It is aggressive, progressive, and generally diagnosed at late stage. The metastases to kidney, peritoneum, and subcutaneous tissue have been described in the literature [7]. Bansal et al. reviewed SEER database for evaluation of outcome of patients with cervical cancer based on different histological types. Five-year survival for stages IB, III, and IV was 80%, 32%, and 17% for squamous cell carcinoma. The patients with adenocarcinoma of stages IB, III, and IV had 83%, 19%, and 9% survival rate. Stage IB, II, and IV sarcomas had survival of 67%, 20%, and 11%, respectively. Similar to sarcomas, SSCC is a highly progressive cancer with low survival rate. Five-year survival plummets with increasing stage, from 90% for stage I to <5% for stage IV. Other prognostic factors include degree of differentiation, extent of the carcinomatous component, size of the tumor, and age at presentation [14–16].

Disease-free survival and response to therapy also vary by stage. Brown et al. reported complete response to first-line therapy in nine patients with SSCC with median disease-free survival of 4.9 months. Two patients, with stage I and II disease, were disease-free for 40 months. After relapse, none of the patients responded to second-line therapies of radiation, surgery, and chemotherapy.

Our patient had a similar course for her stage IIb cancer. She was disease-free for at least four months before being lost to follow-up. Thirteen months later she presented with extensive metastatic disease that had some response to palliative radiotherapy before opting for hospice.

The most common causes of chest mass in adults are inflammatory or infectious conditions and tumors. More than 50% of tumors are malignant. Primary chest wall tumors account for only 5% of thoracic neoplasms. The most common tumors present as local extension of thoracic tumors including breast, lung, pleura, and mediastinum or metastases from thoracic tumors, renal cell carcinoma, thyroid cancer, colon cancer, and melanoma. Lymphomas and chondrosarcoma account for most cases of malignant neoplasms in chest wall [17, 18]. To our knowledge, this is the first case reporting chest mass secondary to metastasis from SSCC of the cervix.

## 4. Conclusion

In summary, sarcomatoid squamous cell carcinoma of the cervix is a very rare and aggressive cancer. Despite good response to first-line therapy, it tends to recur early and does not respond to second-line therapy. Radiation therapy seems to be the most effective modality for treatment, but randomized controlled trials of therapy are impractical.
